# Block network mapping approach to quantitative trait locus analysis

**DOI:** 10.1186/s12859-016-1351-8

**Published:** 2016-12-22

**Authors:** Zeina Z. Shreif, Daniel M. Gatti, Vipul Periwal

**Affiliations:** 1 Laboratory of Biological Modeling, NIDDK, National Institutes of Health, Bethesda, MD 20892 USA; 2 The Jackson Laboratory, 600 Main Street, Bar Harbor, ME 04609 USA

**Keywords:** QTL mapping, Interval mapping, Bayes’ theorem

## Abstract

**Background:**

Advances in experimental biology have enabled the collection of enormous troves of data on genomic variation in living organisms. The interpretation of this data to extract actionable information is one of the keys to developing novel therapeutic strategies to treat complex diseases. Network organization of biological data overcomes measurement noise in several biological contexts. Does a network approach, combining information about the linear organization of genomic markers with correlative information on these markers in a Bayesian formulation, lead to an analytic method with higher power for detecting quantitative trait loci?

**Results:**

Block Network Mapping, combining Similarity Network Fusion (Wang et al., NM 11:333–337, 2014) with a Bayesian locus likelihood evaluation, leads to large improvements in area under the receiver operating characteristic and power over interval mapping with expectation maximization. The method has a monotonically decreasing false discovery rate as a function of effect size, unlike interval mapping.

**Conclusions:**

Block Network Mapping provides an alternative data-driven approach to mapping quantitative trait loci that leverages correlations in the sampled genotypes. The evaluation methodology can be combined with existing approaches such as Interval Mapping. Python scripts are available at http://lbm.niddk.nih.gov/vipulp/. Genotype data is available at http://churchill-lab.jax.org/website/GattiDOQTL.

**Electronic supplementary material:**

The online version of this article (doi:10.1186/s12859-016-1351-8) contains supplementary material, which is available to authorized users.

## Background

Quantitative variations in living organisms result from environmental factors and multiple segregating genes [1]. The search for genomic markers that are linked to quantitative traits is an important first step towards finding the gene variants responsible for the observed phenotype, and is consequential for commercial breeding purposes and for uncovering the mechanistic underpinnings of pathologies. Linkage between genetic loci and morphological traits was first demonstrated almost a century ago [2] but early efforts [3, 4] were difficult due to the sparsity of known genetic markers across the entire genome.

The mapping problem for quantitative trait loci (QTL) is, briefly stated, to find the genetic markers that correlate with measured quantitative traits. Single marker regression [2, 5] was the traditional approach to mapping quantitative trait loci. This one-by-one analysis has well known drawbacks e.g. effect size is confounded with marker separation [6]. The availability of dense genetic linkage maps ushered in modern quantitative genetics [7–9] and single marker regression has been superseded by interval mapping (IM) [10–13]. IM allows a more accurate determination of the location and effect size of a QTL as the likelihood of a QTL can be placed in the context of its genomic position. It still maps only a single locus at a time, contradicting the known polygenic character of quantitative traits.

This led to the formulation of multiple IM methods and composite IM with the introduction of markers used as covariates [14–19]. The issue of the selection of the appropriate covariates remains an interesting challenge, and the genomic context of a trait is not as clear as with single IM. The present paper is directly comparable only to standard single IM.

The use of linkage maps, obtained using multi-point analysis of marker segregation data, is a major advantage of these IM methods compared to single marker regression, but is considered as a separate preliminary step before IM. In the present work, we report on a method, Block Network Mapping (BNM), that incorporates linkage through an experimental data-driven linkage network found using Similarity Network Fusion (SNF; [20]) combined with a new Bayesian approach to locus selection. Ref. [20] did not suggest this novel application of SNF.

To develop BNM, we used synthetically generated phenotypes paired with real genotypes obtained in a study of white blood cells [21] in a specific strain of mice, Diversity Outbred (DO)[22] mice. These were developed to overcome the low mapping resolution of conventional mouse crosses. As an example, [23] demonstrated that behavioral traits could be mapped with high precision with even a modest number of animals.

We investigated the effect size and population size dependence of the false discovery rate (FDR), the power, and the receiver operating characteristic (ROC) obtained using our method, BNM, compared to the standard expectation maximization (EM) implementation of IM implemented in the R/qtl package [24].

## Methods

**Fig. 1:**
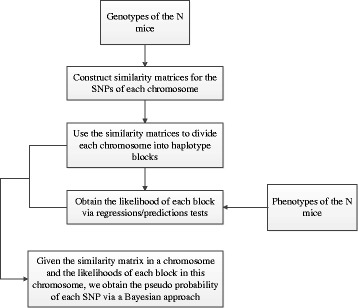
Flowchart outlining the major parts of the BNM algorithm

The BNM algorithm can be divided into three major parts outlined in Fig. 1. For any finite sample of genotypes, there are correlations between genotype markers due to the finite amount recombination that could have occurred. Our approach is to first find contiguous blocks of markers in a data-driven but phenotype-independent manner, which we term haplotype blocks. The idea of looking for such blocks is inspired by multiple IM [14–19] though our approach to finding these blocks is based on ideas from [20]. They are defined by clustering the SNPs based on similarity matrices constructed from the information obtained from the genotypes of the *N* subjects being studied (Section “Obtaining the haplotype blocks”). Second, we compute the likelihood of each block, i.e., the likelihood that there is at least one marker in this block that is contributing to the overall phenotypic effect (Section “Obtaining the block likelihoods”). Third, using the similarity matrices and likelihoods obtained in the first two parts, we calculate the empirical likelihood, of each marker via a Bayesian approach (Section “Obtaining the SNP empirical pseudo-probabilities”), considering each marker as a possible ‘model’ of the phenotype data.

### Genotype data

The real genotype data [21] in a specific strain of mice, Diversity Outbred (DO)[22] mice, underlying the simulated phenotypes that were used to develop and test our approach is available at http://churchill-lab.jax.org/website/GattiDOQTL.

### Obtaining the haplotype blocks

We first represent each sequence of SNP pairs as a sequence of four numbers: 0 (when the SNP is composed of two dominant alleles), 1 (when the SNP is composed of one dominant and one recessive allele), 2 (when the SNP is composed of two recessive alleles), and 3 (when the value of the SNP is missing). We remove SNPs missing values on more than half of the mice and then use SNF to cluster the remaining SNPs based on both their distance matrix *D*, and the mutual information matrix *I*.

#### Distance matrix

We define a distance matrix $D_{ss^{\prime }}$ between SNPs *s* and *s* ^′^ by using the genetic distance between the SNPs, measured in centiMorgans:

1 $$  {D}_{ss^{\prime}} = |\text{cM location of SNP } {s} - \text{cM location of SNP } s^{\prime}|  $$

We also tried using the actual base position index along a chromosome to define the distance matrix, but the final results were not much different.

#### Mutual information matrix

We suppose that the phenotypes measured have been transformed into positive values by exponentiating. If *w*_*m*_ is the phenotype of mouse *m*, we define a normalized phenotype *ρ*_*m*_ by $ \rho _{m} = {w_{m}}/{\sum _{m^{\prime }}{w_{m^{\prime }}}}.$ By definition, $\sum _{m}\rho _{m} = 1.$ With the possible values of any SNP *s* taking values *α*=0,1,o*r* 2, the phenotype-weighted mutual information between two SNPs *s* and *s* ^′^ is defined as

2 $$  \check{I}_{ss^{\prime}} = \sum_{\alpha\beta} {P_{s\alpha s^{\prime} \beta}\log \left[\frac{P_{s\alpha s^{\prime} \beta}}{P_{s\alpha}P_{s^{\prime} \beta}}\right]}  $$

where $ P_{s\alpha s^{\prime } \beta } = \sum _{m} {\rho _{m} \sigma _{m,s_{\alpha }} \sigma _{m,s^{\prime }_{\beta }}} $, $ P_{s\alpha } = \sum _{s^{\prime } \beta } {P_{s\alpha s^{\prime } \beta }} = \sum _{m} {\rho _{m} \sigma _{m,s_{\alpha }}} $, and

3 $$ \sigma_{m,s_{\alpha}} = \left\{ \begin{array}{ll} 1 & \text{when SNP } {s} \text{ of mouse } {m} \text{ is in state } \alpha,\\ 0 & \text{otherwise.} \end{array}\right.  $$

Note that $ \sum _{\alpha \beta }{P_{s\alpha s^{\prime } \beta }} = \sum _{m}{\rho _{m} \sum _{\alpha \beta } {\sigma _{m,s_{\alpha }} \sigma _{m,s^{\prime }_{\beta }}}} = \sum _{m}{\rho _{m}} = 1.$ When the value of the SNP is missing (i.e., when it is in state ‘3’), it is randomly assigned a state 0, 1, or 2 with a probability equal to the distribution of each state for this SNP among the subjects with available data. We want a sample-driven measure of the mutual information between SNPs that is independent of the phenotype. We could, of course, simply take the phenotype to be unity for all *m*, but in order to avoid bias due to the empirical distribution of phenotype values, we permute the phenotypes to obtain a phenotype-independent mutual information by averaging $\check {I}$ over a large number of permutations over the phenotype values of the subjects,

4 $$  {I}_{ss^{\prime}} = \sum_{perm} {\check{I}^{perm}_{ss^{\prime}} / N_{perm}}  $$

where *ρ* ^*perm*^ is a permutation of $ \rho, \check {I}^{perm}_{ss^{\prime }} $ is the same as $\check {I}_{ss^{\prime }} $ but with *ρ* ^*perm*^ replacing *ρ*, and *N*_*perm*_ is the total number of permutations. Note that with every permutation, the missing values of SNPs are randomly assigned a state independent of the previous permutation. In this way, our empirical *I* is independent of the actual phenotypes, but may possibly depend on the distribution of phenotype values. In more detail, suppose that the samples are drawn with unknown bias. Then, a uniformly weighted mutual information between SNPs would be a biased estimator of SNP-SNP mutual information, due to the unknown sampling bias in the observed samples. For example, if the phenotype values are skewed due to sampling bias, our empirical permutation formulation of *I* will maintain the skewed distribution, while reducing the effect of biased sampling on the estimated SNP-SNP mutual information. This may reduce the power to detect correlations, but it will not enhance correlations due to biased sampling, so this is a conservative approach. Permutation tests are often used in similar settings in QTL analysis [25].

#### Similarity matrix

The matrices *I* and *D* defined in this manner are sample-dependent and sample-independent, respectively. Moreover, the mutual information similarity is in no way constrained by contiguity on the chromosome, and indeed, the two similarity measures are defined in units that are not directly comparable. We want to find a principled approach to combining these similarity measures into a single unified similarity matrix. The Similarity Network Fusion (SNF) approach [20] is a recently published algorithm that solves exactly this problem, by translating each independent similarity measure separately into a network, and then fusing these networks into a combined single network. An important point emphasized in Ref. [20] is that SNF is an algorithm for fusing network information obtained from many different data types characterizing a group of subjects into a combined similarity network, even for data types as different as methylation data and expression data. For example, the similarity metric suggested in Ref. [20] (Online Methods Section) is chi-squared distance for discrete variables and agreement-based measure for binary variables, compared to Euclidean distance for continuous variables. This versatility makes SNF particularly well-suited to our application. Thus, for each chromosome with SNF, we obtain a similarity matrix of SNPs which is defined by a fusion of the distance matrix *D* and the mutual information matrix *I*. Fusion using the SNF algorithm requires specifying two parameters: the number of neighbors *κ* and hyperparameter *η*. We elaborate on the choice of these parameters in the next subsection.

#### Hierarchical clustering

Given the fused similarity matrix, we use it to find blocks of SNPs that are correlated in the available dataset independent of the phenotype and, due to the use of the genetic distance matrix *D*, contiguous on the chromosome. A clustering method must be chosen to carry out this block decomposition based on the fused similarity matrix. We implemented a version of hierarchical clustering, and as in most approaches to defining clusters with hierarchical clustering, we must specify how the tree is used to define the final clusters.

**Fig. 2:**
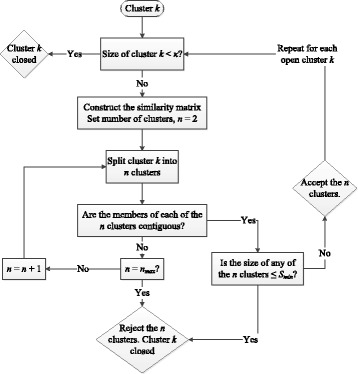
Flowchart outlining the Hierarchical Clustering algorithm

We separate the SNPs into different clusters based on their fused similarity matrix in the following manner. We perform hierarchical clustering where we iteratively divide a cluster into two clusters, or more if the binary split did not satisfy the conditions described below (see Fig. 2. This will form an *n*-ary tree (i.e., with *n* branches emanating from the end of each parent branch) where the end branches are the final clusters to be used. This turns out to be mostly a binary tree for the present dataset. At each iteration, the splitting process is repeated for every “open” branch. An open branch is one that did not meet the stopping conditions. If a branch meets all the stopping conditions then the branch will be considered “closed”. For each open branch/cluster *k*, we first check if the size of cluster *k*, *S*_*k*_, is smaller than *κ*. If it is, then branch *k* ends, i.e. cluster *k* is now closed. Otherwise, the splitting process starts: first, the similarity matrix for cluster *k* is constructed. Then, starting with setting the number of clusters *n* to *n*=2, cluster *k* is split into *n* new clusters via spectral clustering. If all the new clusters have contiguous members and none of them is smaller than a minimum size, *S*_*min*_ (here *S*_*min*_=2), then the new clusters are accepted and considered open, i.e., they move on to the next iteration. If these conditions are not met, we incrementally increase *n* and repeat the splitting test until either the new clusters are accepted or *n* reaches a maximum number *n*_*max*_ in which case the new clusters are rejected and branch *k* ends. This process is illustrated in Fig. 2. Note that *n*_*max*_ depends on the size of the cluster being split, *n*_*max*_= min(*S*_*k*_/*S*_*min*_,*t*_*max*_) where *t*_*max*_ is the maximum number of iterations allowed. The iterations stop when either all branches are closed or the maximum number of iterations is reached.

We still need to decide the values of *κ* and *η*, as the final clustering results will differ depending on these values. Therefore, we perform the above hierarchical clustering algorithm with different values of *κ*,*η* pairs (*κ*=10,11,…15 and *η*=0.3,0.4,…0.7). The optimal *κ*,*η* pair is the one that leads to the smallest cluster sizes, as we wanted to obtain higher resolution for the correct SNP. Note that in some examples where we selected for larger clusters, we found a tradeoff between SNP localization and phenotype prediction accuracy. As we focus in this paper on the SNP mapping problem, we chose smaller cluster sizes. In particular, we look at the biggest cluster at the end branches and choose the *κ*,*η* pair with the smallest biggest cluster (the minimax criterion in this context). If the size of these smallest biggest clusters is the same then we compare the number of big versus small clusters.

### Obtaining the block likelihoods

The likelihood of a block is the likelihood that at least one SNP in this block contributes to the overall phenotype value. If a block has an effect on the phenotype, then a regression model on this block should have a good predictive power relative to a null model (see Section “The relative predictive powers of the blocks” below). If we assume that only one block in each chromosome contributes to the overall phenotype value, then the likelihood of a block should be obtained by comparing the relative predictive power of all the blocks in the chromosome in which the block resides (see Section “The likelihoods of the blocks” below).

#### The relative predictive powers of the blocks

For each block *k*, we perform *N*_*trial*_ trials (*N*_*trial*_=1000) of regression/ testing simulations. For each trial *t* we randomly divide the data points into two equal halves, a training and a testing set. A data point is composed of a subject’s phenotype value and its block *k* genotype, i.e., its sequence of SNP states composing block *k*. Then, we test two models, one to obtain the predictive power of block *k* for trial *t* and another to serve as the null model for block *k* and trial *t*. For both models, we use the sequence phenotype inference approach described in [26]. This approach allows the investigation of possible nonlinear dependence of the phenotype on allele frequency.

For the first model, we train its parameters on the data points in the training set and then use it to predict the phenotypes of the subjects in the test set. Comparing our predicted phenotypes, $ {w^{pred}_{k,t}}\phantom {\dot {i}\!}$, to the actual values of the phenotypes in the test set, $ {w^{test}_{t}}\phantom {\dot {i}\!}$, we obtain the Pearson correlation *r*_*k*,*t*_ between $ \log ({w^{pred}_{k,t}})\phantom {\dot {i}\!}$ and $ \log ({w^{test}_{t}})\phantom {\dot {i}\!}$ for trial *t*, block *k*. The sign of the SNP’s effect on the phenotype never appears in these calculations because we are always comparing the predicted phenotype values with the test set phenotype values. If the prediction is correct, whether the SNP enhances or decreases a phenotype, the value of *r*_*k*,*t*_ will be positive.

For the second (null) model, we perform *N*_*p*_ permutation trials [25]. For each permutation trial *p*, we permute the phenotypes of the data points in the training set before training the model parameters. Then, similar to the first model, we predict the phenotypes of the subjects in the test set to obtain the Pearson correlation $ r^{p}_{k,t}\phantom {\dot {i}\!}$ between $ \log ({w^{pred,p}_{k,t}})\phantom {\dot {i}\!}$ and $ \log ({w^{test}_{t}})\phantom {\dot {i}\!}$, where $ {w^{pred,p}_{k,t}}\phantom {\dot {i}\!}$ is the set of predicted phenotypes using the training model for block *k*, from trial *t* and permutation trial *p*. The relative predictive power of block *k* for trial *t* can now be defined via the ratio

5 $$  R_{k,t} = \frac{\exp \left(r_{k,t}\right)}{\sum_{p}{\exp \left(r^{p}_{k,t}\right)}}.  $$

**Fig. 3:**
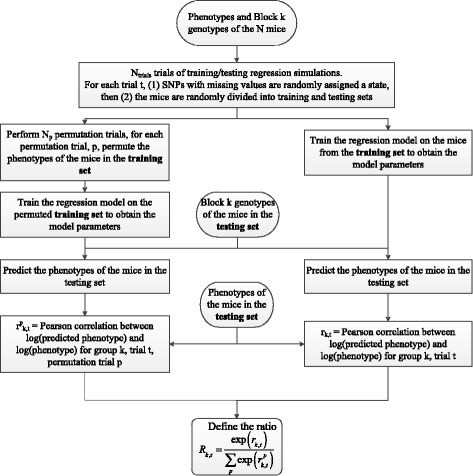
Flowchart outlining the algorithm steps to obtain the ratio in Eq. 5

This algorithm is outlined in Fig. 3. Notice that the exponentiation of the Pearson correlations here implies that possible negative values of *r*_*k*,*t*_ or $r^{p}_{k,t}$ lead to lower values, as is appropriate.

#### The likelihoods of the blocks

We can finally define the likelihood *L*_*k*_ of block *k* as the fraction of trials where $ R_{k,t} \geq R_{k^{\prime },t} $ for all *k* ^′^≠*k*.

### Obtaining the SNP empirical pseudo-probabilities

In formulating our approach in a Bayesian setting, we consider each SNP as a possible model for the observed phenotype. In particular, we assume that each chromosome has only one possible ‘true’ SNP. Our prior probability is that every SNP is equally likely to be causative. It remains then to define the likelihood of the ‘data given the model’ part of the Bayes computation, which in our context corresponds to ‘the phenotypes observed given the causative SNP *s*’, to find the probability of the SNP *s* as the model given the phenotype data, as is standard in applications of Bayes’ theorem.

To motivate our likelihood function for the data given the model, we first note that a higher likelihood value of a block *k*, *L*_*k*_, suggests that at least one SNP in block *k* is contributing to the quantitative phenotype. As discussed above, these blocks are chosen such that SNPs in the same block are more correlated to each other than to SNPs in a different block. However, there are still correlations between SNPs from different blocks, albeit not strong enough to be included in the same block. Because of these inter-block correlations, we expect that even blocks that do not contain the causative SNP could have a high likelihood as well simply due to correlations that exist in the finite set of sampled genotypes, and we can quantify this as follows. Assuming that there is only one causative SNP on each chromosome, if SNP *s* is the one then the likelihood that a block *b* will show an effect should be proportional to the odds ratio, $ L^{0}_{s,b}\phantom {\dot {i}\!}$ of the correlation between SNP *s* and block *b* compared to its correlation to all other blocks in the same chromosome. We define

6 $$ L^{0}_{s,b} = Q_{s,b} \bigg/ \sum_{b^{\prime} \neq b} {Q_{s,b^{\prime}}},  $$

where $ Q_{s,b} = \max _{s^{\prime } \in b, s^{\prime } \neq s}{M_{s,s^{\prime }}}$, and *M* is the similarity matrix for the corresponding chromosome obtained by fusing its distance and information matrices as described in Section “Hierarchical clustering” using the optimal *κ*,*η* pair values obtained while performing the hierarchical clustering.

Note that $L^{0}_{s,b}\phantom {\dot {i}\!}$ is phenotype independent (as we permuted the phenotypes in computing *I*) and is simply a measure of the correlation between SNP *s* and block *b* based on the finite amount of data available. We also tried using *L* ^0^ defined in terms of the mean instead of the maximum of *M*, but this did not materially affect the results.

Using the Pearson correlation again but in a completely different context, we use our definition of $L^{0}_{s,b}\phantom {\dot {i}\!}$ to define the empirical likelihood of a SNP *s* given the data as the Pearson correlation, *r*_*s*_, between the sequence of phenotype-independent likelihoods $ { L^{0}_{s,1},L^{0}_{s,2},\ldots L^{0}_{s,N_{c}}} \phantom {\dot {i}\!}$ and the sequence of phenotype-dependent likelihoods (section “The likelihoods of the blocks”) $ { L_{1},L_{2},\ldots L_{N_{c}} },\phantom {\dot {i}\!}$ where *N*_*c*_ is the number of blocks in chromosome *c* which contains the SNP *s*. If this Pearson correlation *r*_*s*_ is negative, we define *r*_*s*_≡0. It should be emphasized here that *r*_*s*_<0 does not correspond to a SNP that has a negative correlation with the phenotype. What is being compared here is the correlation pattern between likelihoods of SNP blocks, phenotype-independent (which indicates genetic linkage independent of phenotype considered) and phenotype-dependent (which indicates linkage weighted by phenotype). A negative correlation *r*_*s*_<0 occurs when the genetic linkage of SNP blocks is exactly the opposite of the linkage suggested by the phenotype weighting. For small sample sizes, such negative correlations can appear by chance, but just as in the definition of *r*_*k*,*t*_ in Section “The relative predictive powers of the blocks”, they are not related to the sign of the effect of the SNP block on the phenotype. We call this empirical likelihood, *r*_*s*_, the pseudo-probability of *s* because it takes values between 0 and 1 as defined, with the ‘pseudo-’ prefix to emphasize that it is not, in fact, a probability. We will use *R*(*s*)≡1−*r*_*s*_ in our calculations of power, false discovery rate and other measures of our methodology.

### Summary

As we have given several definitions in the preceding Method subsections, we summarize the relevant information to make the “Results” Section clearer in conjunction with 1.

- *L*_*k*_: The likelihood that a block *k* contains a SNP which has an effect on the phenotype, calculated using training/testing splits of the data and null trials with permuted training phenotypes.
- $L^{0}_{s,k}:$ The phenotype-independent likelihood that a SNP *s* is correlated with a block *k*, calculated from the SNF fused similarity matrix.
- *r*_*s*_: The Pearson correlation coefficient over all blocks *k* between *L*_*k*_ and $L^{0}_{s,k}.$
- *R*: For each SNP *s*, *R*(*s*)≡1− max(0,*r*_*s*_).*R* is defined like this so that increasing FDR *P*-value thresholds correspond to increasing *R*-value thresholds.

## Results

We demonstrate our method by applying it to simulated data with 742 genotypic sequences of Diversity Outbred (DO) Mice and 1000 phenotypes. We simulated phenotypes on the 19 autosomes and did not add a sex effect. We selected 19 genomic locations, one on each autosome, and generated 19 QTL effect sizes from an exponential distribution. Using the genotypes at each location, we created the QTL effects and scaled the variance to be 1. Then we added N(0,1) noise and the QTL effects together.

**Fig. 4:**
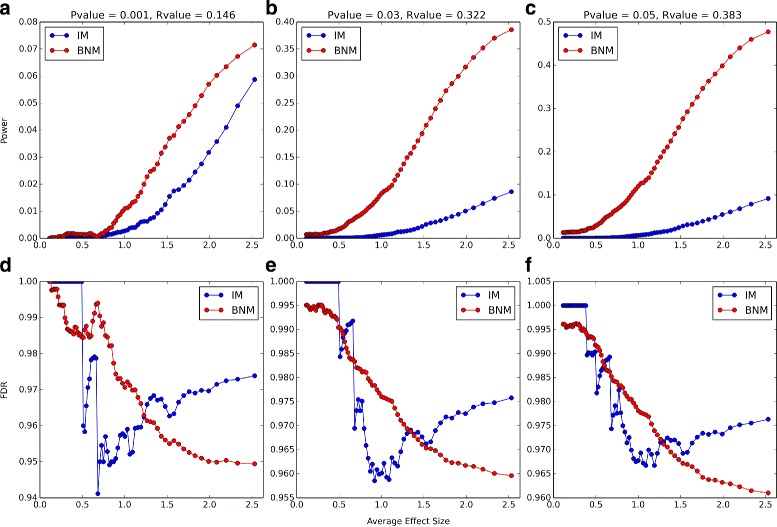
Power and FDR as a function of Effect Size. Power and FDR of the BNM algorithm (*blue*) and IM from the R/qtl package (*red*) with increasing effect sizes. Each point corresponds to the Power (**a**-**c**) or FDR (**d**-**f**) within a group of 4000 data points with an average effect size in the *x-axis*. We show the power and FDR at three *P*-value (for IM) and *R*-value (for BNM) thresholds: 0.001 and 0.146 (**a**, **d**), 0.03 and 0.322 (**b**, **e**), and 0.05 and 0.383 (**c**, **f**). These *P*-value, *R*-value pairs are matched so that they have the same FDR averaged over all points (see Additional file 1: Figure S2)

We compare our results to that of simulations using Interval Mapping (IM) with expectation maximization from the R/qtl package. For the R/qtl simulations, we use scanone with the default settings and calc.genoprob with step = 0 and error.prob = 0. For the *P*-values calculations we run scanone with 1000 permutations (n.perm = 1000). The simulated data are obtained by choosing only one SNP that influences a particular phenotype on each of the 19 autosomes with varying effect sizes. These effect sizes range between 1.65×10^−5^ and 10.03 (see Additional file 1: Figure S1). We compare the power [5, 27, 28] of our method with that of IM for different effect sizes. With 1000 phenotypes, 19 autosomes, and 1 “true signal” on each autosome, we have 19,000 effect size data points. We arrange them in order of increasing effect size and then divide them into 76 groups of 4000 data points with 200 points offset. For example, the first group is composed of the first 4000 data points with the lowest effect sizes, and then the second group is composed of data points 200 to 4200, and so on. Then the power and false discovery rate (FDR) are calculated within each group separately (Fig. 4). While the R/qtl scanone implementation of IM assigns a *P*-value for each SNP, BNM assigns a empirical likelihood *r*_*s*_ (as described in the “Methods” Section) and thus an *R*-value = 1−*r*_*s*_. To compare the power of the two methods at matching thresholds, we choose a *P*-value for IM and look for the BNM *R*-value with comparable average FDR over the 76 groups (Additional file 1: Figure S2). We compare the power of our method with that of IM at 3 different *P*-value thresholds (*P*-value = 0.001, 0.03, 0.05) and their FDR-matching BNM *R*-values (*R*-value = 0.146, 0.322, 0.383). In all cases BNM has a higher power (Fig. 4 a–c). This is more prominent at higher effect sizes even though BNM has a monotonically decreasing FDR with increasing effect size (Fig. 4 d–f).

**Fig. 5:**
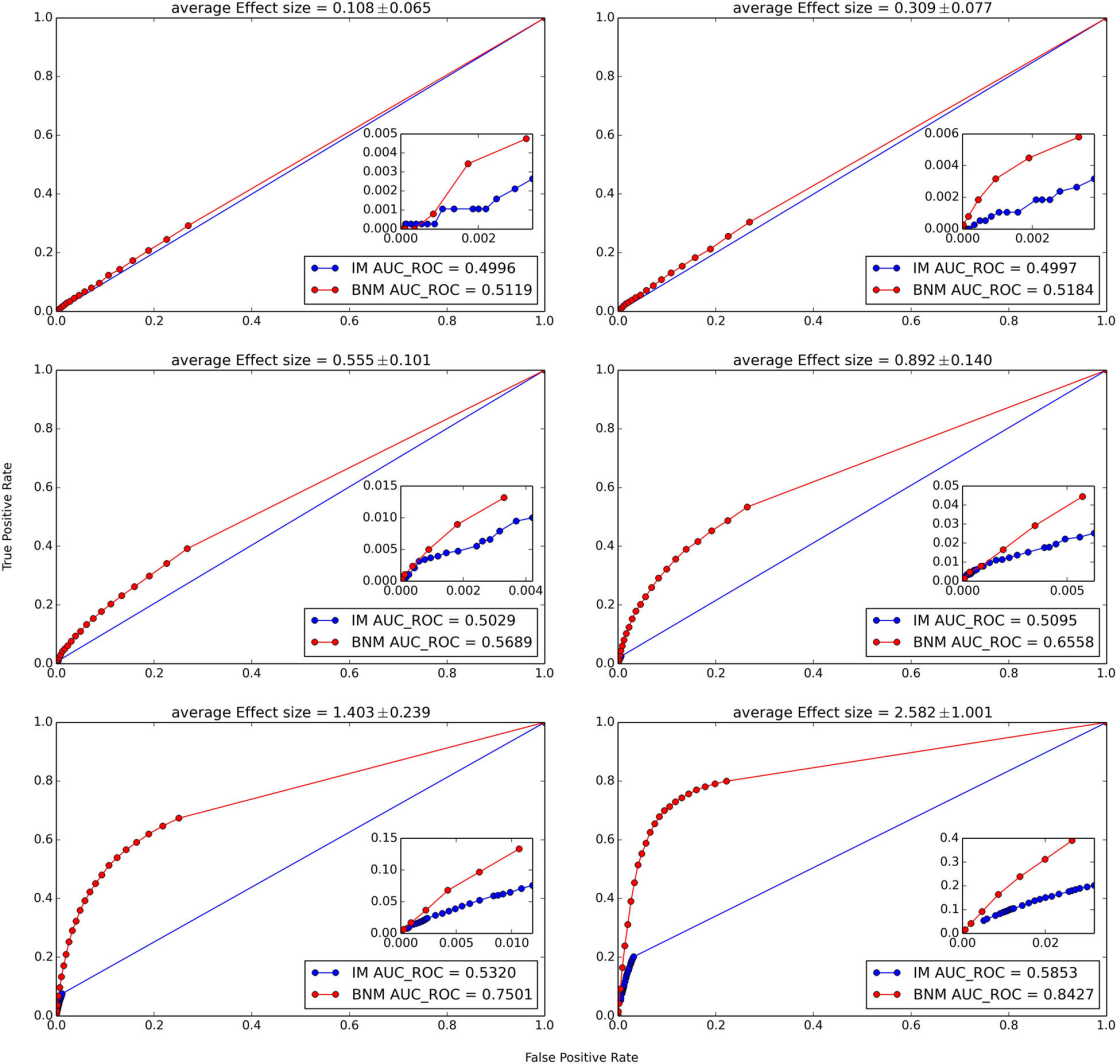
ROC curves as a function of Effect Size. ROC curves for IM (*blue*) and BNM (*red*) within 6 groups of 3800 data points with average effect sizes 0.108±0.065,0.309±0.077,0.555±0.101,0.892±0.140,1.403±0.239 and 2.582±1.00

We also compare the ROC curves in 6 different effect size ranges (Fig. 5). In this case the data points are divided into 6 groups of 3800 data points each with an offset of 3100 points. BNM outperforms IM at moderate and high effect sizes. At very low effect sizes, both IM and BNM do not have much predictive power.

In all of the above results, the power and FDR calculations are based on the precise location of the true SNP. In other words, even if the method predicts a signal (at a specified threshold) in the neighborhood of the “true signal”, it is considered a false positive. This is more stringent than the usual expectation of QTL mapping [29] so we repeated the above analysis after uniformly dividing each autosome into blocks of SNPs within *d* Mb from each other. For example, if one or more signals are obtained in a particular block, this counts as 1 true positive (if the true signal is in this block) or 1 false positive (if the true signal is not in this block). We did the analysis at three different block sizes, *d*=2 (Additional file 1: Figures S3, S4 and S5), *d*=3 (Additional file 1: Figures S6, S7 and S8), and *d*=4 (Additional file 1: Figures S9, S10 and S11). BNM still shows a higher power than IM at all block sizes and (*P*-value, *R*-value) thresholds, despite the fact that adding this freedom improves the IM R/qtl results much more than it does the results from BNM. Even with the use of blocks of varying sizes, IM shows a decreasing and then increasing FDR as effect sizes are increased while the BNM FDR continues to be a monotonically decreasing function of effect size (Additional file 1: Figures S3, S6 and S9).

**Fig. 6:**
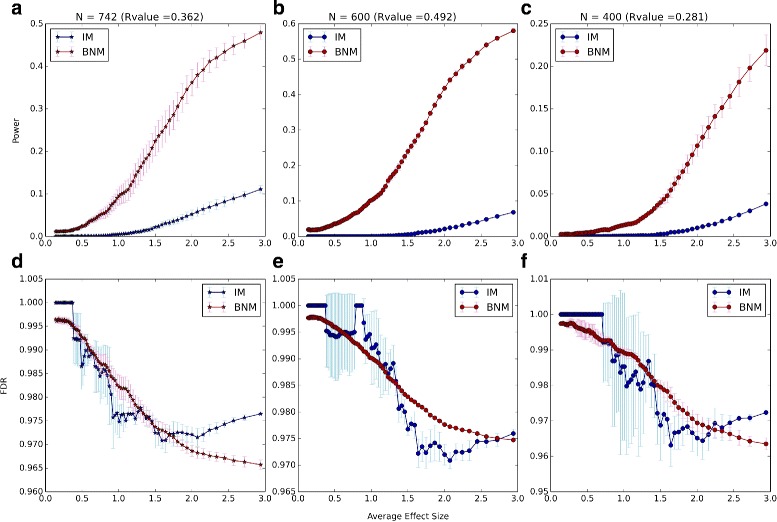
Power and FDR as a function of Sample Size. Power and FDR of the BNM algorithm (*blue*) and IM from the R/qtl package (*red*) with increasing effect sizes. Each point corresponds to the Power (**a**-**c**) or FDR (**d**-**f**) within a group of 2000 data points with an average effect size in the *x-axis*. We show the power and FDR at *P*-value = 0.05 (for IM) and the matching BNM *R*-value such that IM and BNM have the same FDR averaged over all points (see Fig. 7). In (**a**,**d**) we use all the mice (Nmice = 742) and three samples of 500 phenotypes from the 1000 simulated phenotypes; the FDR matching *R*-value = 0.362 (see Fig. 7 a). In (**b**, **e**) we use three samples of randomly selected 600 mice out of the 742 mice available; the FDR matching *R*-value = 0.492 (see Fig. 7 b). In (**c**, **f**) we use three samples of randomly selected 400 mice out of the 742 mice available; the FDR matching *R*-value = 0.281 (see Fig. 7 c). The plots are the means over the three samples in each case, and the errorbars are the standard deviations from the mean in each case

**Fig. 7:**
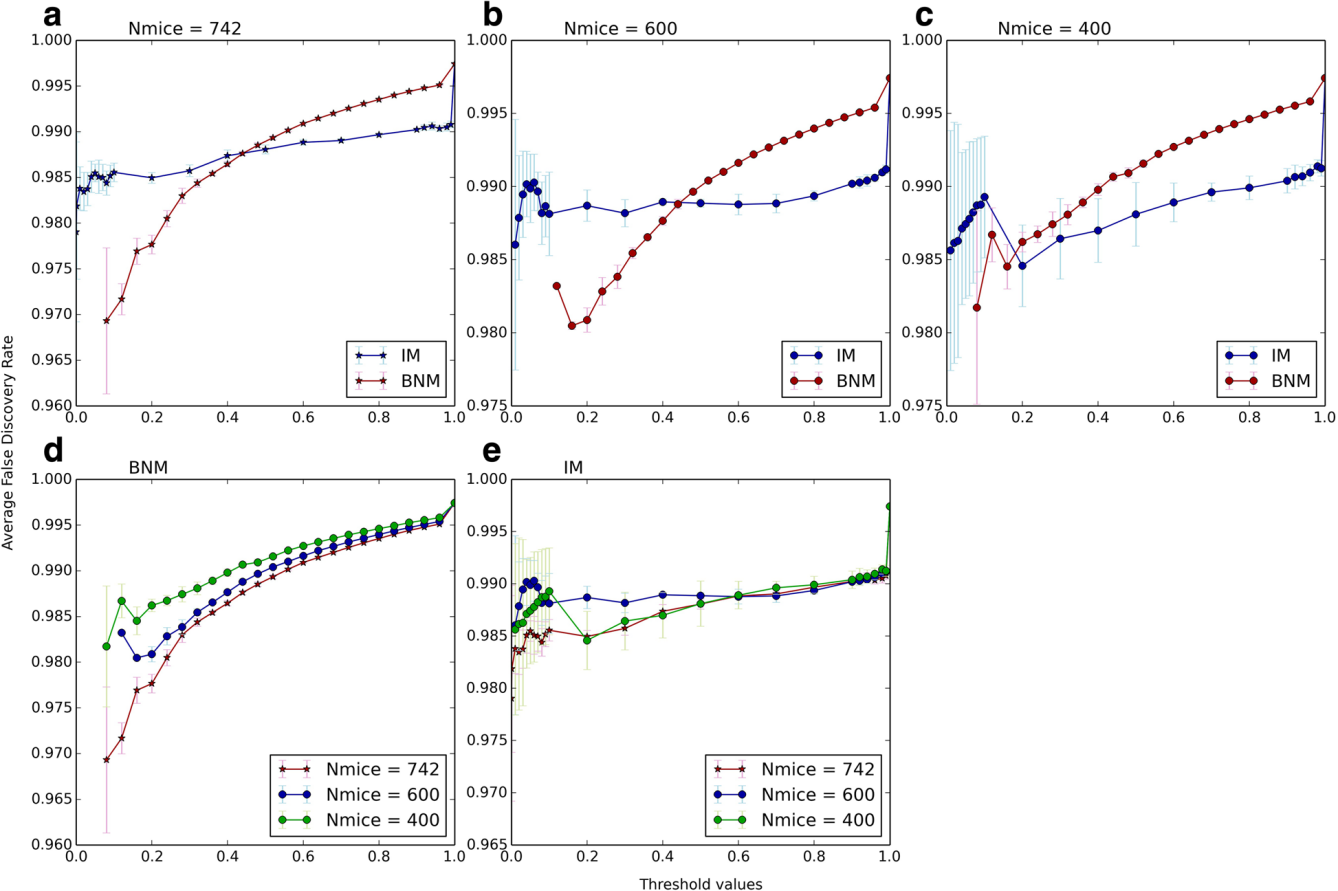
False Discovery Rate variability. The average false discovery rates (*y-axis*) are the mean over the three samples of the average over the FDRs within each of the 76 effect size groups in Fig. 6 at different *P*-value (for IM (*blue*)) or *R*-value (for BNM (*red*)) thresholds (*x-axis*). In (**a**) we take the average FDR over the points in Fig. 6 d for each of the three samples. The plot is mean over the three samples and the errorbars are the standard deviation from the mean. Similarly we take the average over the points in Fig. 6 e (**b**), and Fig. 6 f (**c**). In (**d**) we replot all together the results for BNM (*red plots* in (**a**-**c**)). In (**e**) we replot all together the results for IM (*blue plots* in (**a**-**c**)

Next we examine how the power and false discovery rates change with the choice of different samples of phenotypes and with decreasing number of mice (Fig. 6). To examine the variation with choice of phenotype sets, we use three samples of 500 phenotypes. In two samples we randomly select 500 of the 1000 phenotypes and in the third we select the 500 phenotypes with the highest average effect sizer over the 19 autosomes. As above, we arrange the data points in order of increasing effect size and then divide them into 76 groups of 2000 data points with 100 points offset. Then the power and FDR are calculated within each group separately (Fig. 6 a, d). At low threshold values we see high variation in the average FDR between the samples (Fig. 7 a) which is due to the low number of predicted signals, making for larger statistical uncertainties. Except for the lowest threshold, this variation decreases when the analysis is repeated after setting blocks of size 2 Mb (Additional file 1: Figure S13a), 3 Mb (Additional file 1: Figure S15a), and 4 Mb (Additional file 1: Figure S17a).

The effect of population size on QTL detection has been demonstrated [30, 31], so we investigated the performance of BNM with a change in the number of mice. To examine the change and variation in FDR as we decrease the number of mice, we randomly select three samples of 600 mice and three samples of 400 mice out of the total number of 742 mice. For all of the 6 samples we used the 500 phenotypes with the highest average effect size over the 19 autosomes. Choosing the phenotypes in this manner slightly increases the fraction of high effect signals which will allow us to go to slightly higher average effect sizes in the 76 groups of data points.

As the number of mice decreases, we see more variation in the FDR between the three samples (Figs. 6 d–f), particularly for IM at low *P*-values (Fig. 7 e). As is to be expected, the FDR increases as the number of mice decreases for both IM (Fig. 7 e) and BNM (Fig. 7 d). For each of the 742 (all mice), 600 and 400 mice samples, we match the IM *P*-values to *R*-values of comparable average FDRs and compare the powers at *P*-value = 0.05 (Fig. 6). In all cases, BNM shows higher power and less variation in FDR than IM. Applying the block analysis with *d*=2,3,4 improves the IM FDR and removes the effect of the lower number of mice but this is not as much the case for the BNM FDR (Additional file 1: Figures S13, S15 and S17). In fact, for the block analysis, BNM’s FDR increases with smaller numbers of mice while IM’s FDR is relatively insensitive, making the matching BNM *R*-value much lower at the chosen IM *P*-value. Now when we lower the number of mice to 400, BNM shows less power (Additional file 1: Figures S12, S14 and S16). Overall, however, BNM shows better ROC curves in all cases with and without the block analysis (see Additional file 1: Figures S18, S19, S20 and S21).

**Fig. 8:**
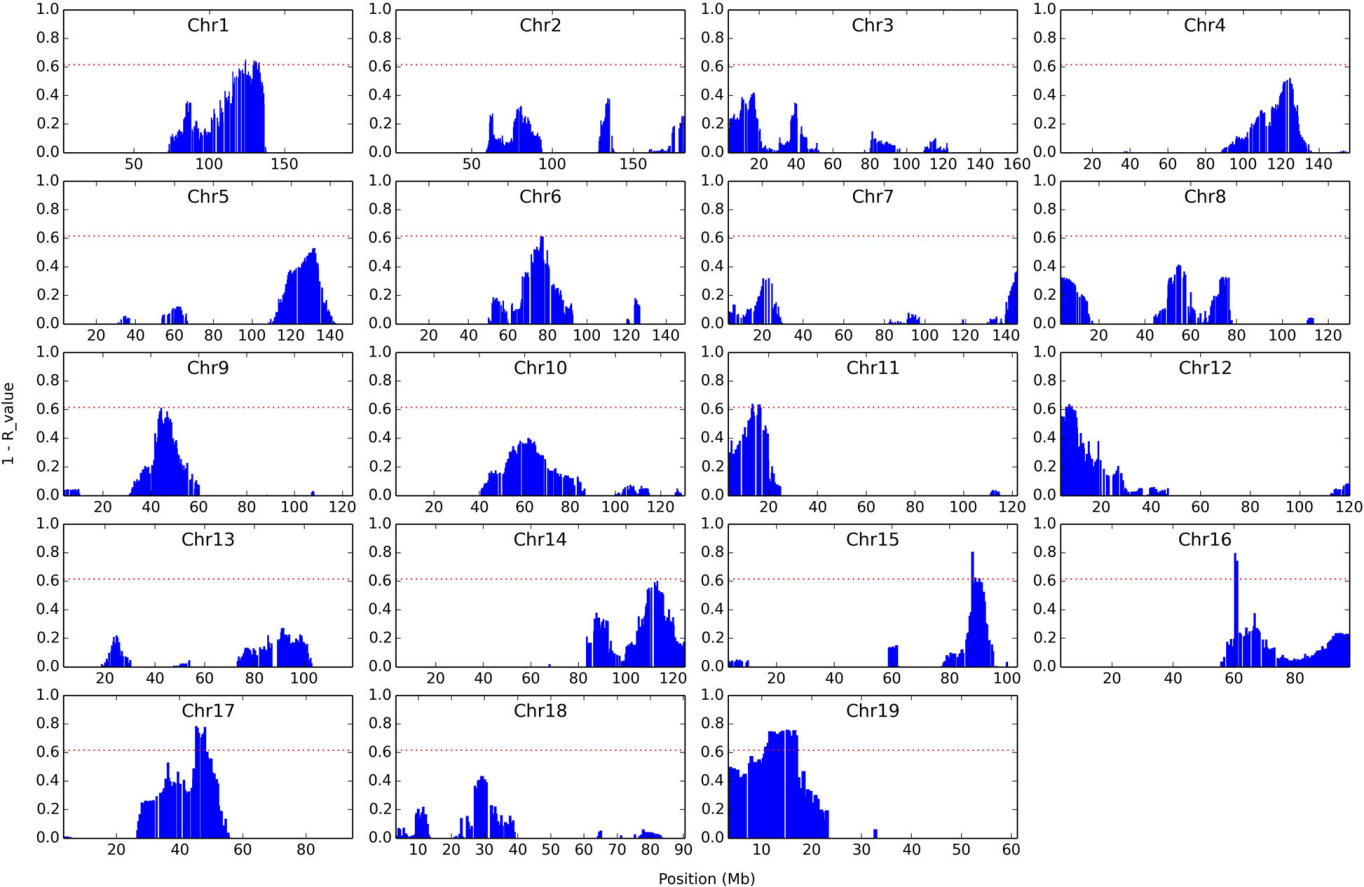
Neutrophil SNP Signals. For each chromosome we show 1 - *R*-value of each SNP (*y-axis*). The *x-axis* shows the location of each SNP in Mb. The *horizontal red dotted line* denotes the threshold value above which signals are detected

Finally, we use the same 742 DO mice to map neutrophil counts in whole blood obtained from [21]. We set our *R*-value threshold to 0.383 since this is the FDR-matching *R*-value to *P*-value = 0.05 in our simulated data. We found signals on loci in chromosomes 1, 11, 12, 15, 16, 17, and 19 (Fig. 8). The loci we found on chromosome 1 are between 123.301336 Mb and 132.515233 Mb. This interval included Cxcr4 which is involved in neutrophil trafficking [32].

## Discussion

We formulated an integrated data-driven approach, Block Network Mapping (BNM), to linkage mapping and phenotype regression using Similarity Network Fusion [20] to define haplotype blocks of SNPs that were then used for phenotype regression. The importance of using SNF or similar network methods is that multiple similarity measures with disparate underpinnings (in our case, genetic distance and empirical mutual information) can be combined using a common graph-theoretic framework which is noise-tolerant. We chose SNF [20] because it is very recent and has proven efficacy. We combined the results obtained from this block-by-block regression with the known inter-block correlations between SNPs using Bayes’ theorem to obtain a final measure of the association of a SNP with the phenotype. Changing *S*_*min*_ to higher values, i.e., using bigger smallest possible clusters did not materially affect our results. However, uniformly segmenting the chromosome into clusters of equal size gave worse results.

QTL locations and effects are specific to populations, and can only be detected when the population is polymorphic at the relevant loci. In light of this, BNM uses no information beyond the genotypes and phenotypes measured in the sample, besides the genetic distance. We did not find much difference between using the genetic distance and the distance measured in base pairs between markers.

We found that the area under the receiver operating characteristic curve (AUROC) exceeded that of IM for all effect sizes, all allowed genome interval sizes (0, 2, 3, 4Mb) and all chosen numbers of mice (742, 600 or 400 total mice). The power of our approach was considerably higher than that of IM in all circumstances, except when we allowed for some genomic distance between the true and predicted SNPs and simultaneously decreased the number of mice to 400. It is known [30, 31] that for QTLs common to two populations, the IM estimate of effect size was reduced in the larger population, supporting the notion that IM overestimates the magnitude of QTL effects in small populations, which may also explain the increase in FDR for IM as sample size is decreased. Power graphs do not directly exhibit the FDR, but the ROC curves show that the predictions made using BNM are more likely to be correct in all circumstances compared to IM. We note that the False Discovery Rates are quite high for this simulated dataset, both for IM and for BNM. As we are presenting our methodology here, it is the relative performance that is of interest.

As our approach works block-by-block, it is somewhat similar to composite IM [14–19] but with a definite prescription for the selection of covariates in the form of SNF clusters. As such, the genomic position of the trait locus is interpretable. Note that accounting for inter-block correlations was crucial for suppressing spurious SNP-phenotype correlations in our approach. However, we have compared our results to IM alone in this paper because composite IM also addresses the presence of multiple loci by partial regression with selected covariate SNPs. A standard approach to composite IM is to add known QTL to the model iteratively, and we can carry this out iteratively as well in BNM, with the QTL uncovered in a first pass with simple IM. However, we are investigating whether a more natural extension of BNM can be developed using the SNF framework.

Our work does not improve on simple IM with respect to effects of opposing sign associated with linked SNPs. It is not clear that our method could be modified to overcome this limitation, though our approach can detect nonlinear dependence on allele dosage. In its present form, we also made the assumption that only one block in a chromosome contributes to the phenotype. This is, of course, an external assumption from the viewpoint of the underlying mathematics. It can be relaxed by using only contiguous parts of chromosomes in the analysis, but these parts will have smaller numbers of blocks, which in turn will lead to lower power. In other words, multiple effects on a chromosome could be detected with BNM albeit with a worse AUROC. It would be more appropriate to investigate better approaches to solving the multiple locus problem [18] within a network paradigm. We are working on extending BNM to account for multiple related phenotypes.

## Conclusions

In this study, we have presented a network approach to QTL analysis that uses sample genotype data to define covariates in a systematic and interpretable manner. Using the network of correlations between SNPs through SNF for finding covariate blocks was a central feature of our approach, along with a Bayesian approach to finding the likely SNPs within blocks using inter-block correlations. Network approaches may be more noise-tolerant and may scale well to larger sets of measured markers.

## Additional file

Additional file 1 Block_Network_Mapping_Supplementary Figures. All supplementary figures referenced in the text, Figures S1-S21. (PDF 35430 kb)

## Abbreviations

AUROC: Area under receiver operating characteristic
BNM: Block network mapping
CIM: Composite interval mapping
cM: centiMorgan
DO: Diversity outbred
EM: Expectation maximization
FDR: False discovery rate
IM: Interval mapping
Mb: Megabase
QTL: Quantitative trait locus
ROC: Receiver operating characteristic
SNF: Symmetric network fusion
SNP: Single nucleotide polymorphism
TPR: True positive rate
