# Supplementary material for: Block network mapping approach to quantitative trait locus analysis
Source: BMC Bioinformatics. 2016 Dec 22;17:544. doi: 10.1186/s12859-016-1351-8 (PMC5178092; doi:10.1186/s12859-016-1351-8)
Supplement: Additional file 1 — Block_Network_Mapping_Supplementary Figures. All supplementary figures referenced in the text, Figures S1-S21. (PDF 35430 kb) [file 12859_2016_1351_MOESM1_ESM.pdf]

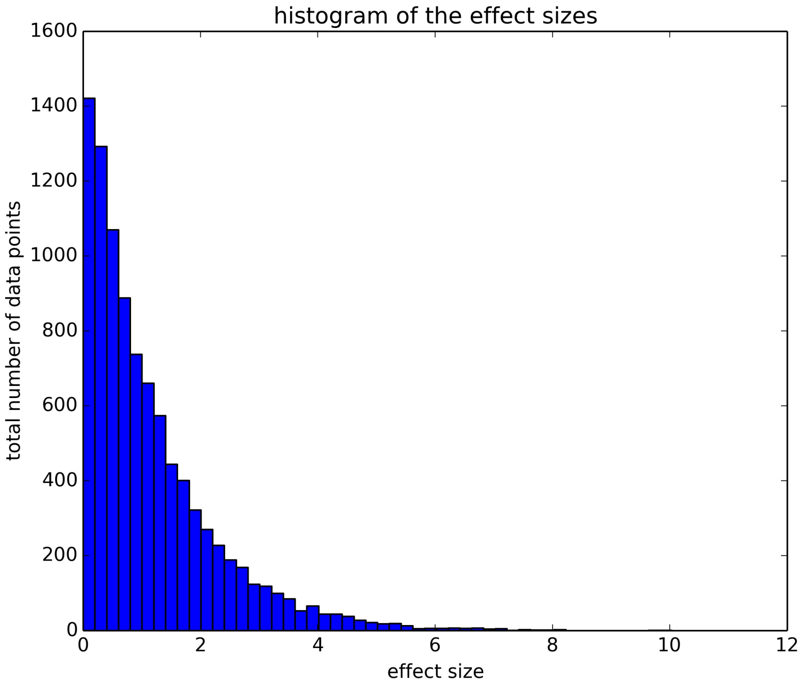

Figure S1: Histogram of the effect size distribution

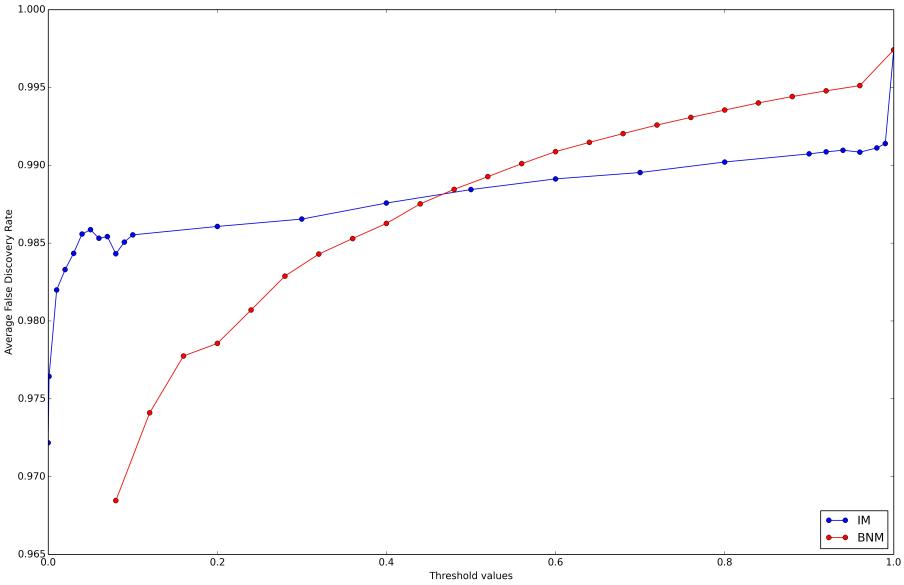

Figure S2: The average false discovery rate (y-axis) is the average over the FDRs within each of the 76 effect size groups in Fig. 4 at different P-value (for IM (blue)) or R-value (for BNM (red)) thresholds (x-axis).

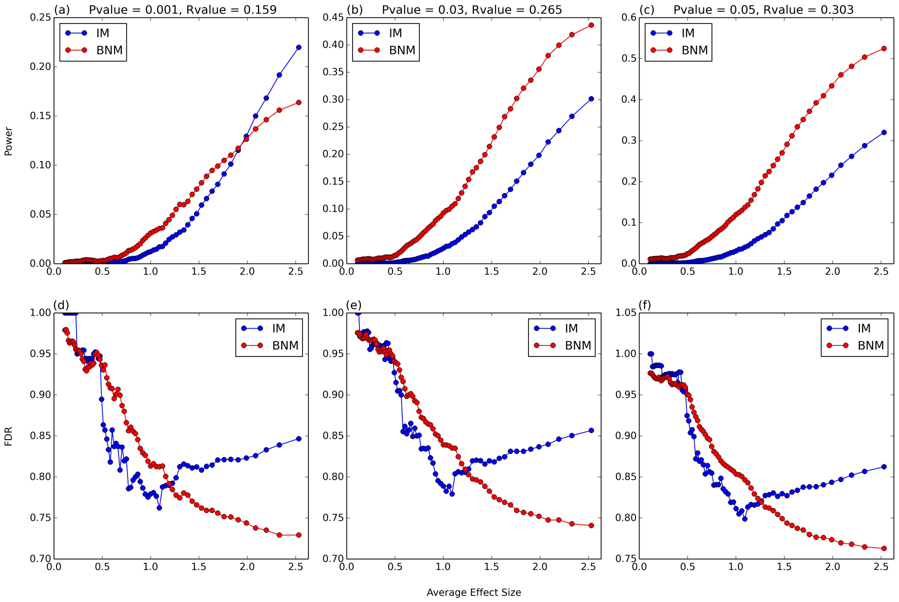

Figure S3: Power and FDR of the BNM algorithm (blue) and IM from the R/qtl package (red) with increasing effect sizes. Here we set 2 Mb blocks. Each point corresponds to the Power (a-b) or FDR (d-f) within a group of 4000 data points with an average effect size in the x-axis. We show the power and FDR at three P-value (for IM) and R-value (for BNM) thresholds: 0.001 and 0.159 (a, d), 0.03 and 0.265 (b, e), and 0.05 and 0.303 (c, f). These P-value, R-value pairs are matched so that they have the same FDR averaged over all points (see Fig. S4). Note that even though at the lowest thresholds (a), when the effect sizes are very high, IM seems to have higher power than BNM. This is actually due to the much higher FDR at these points (d).

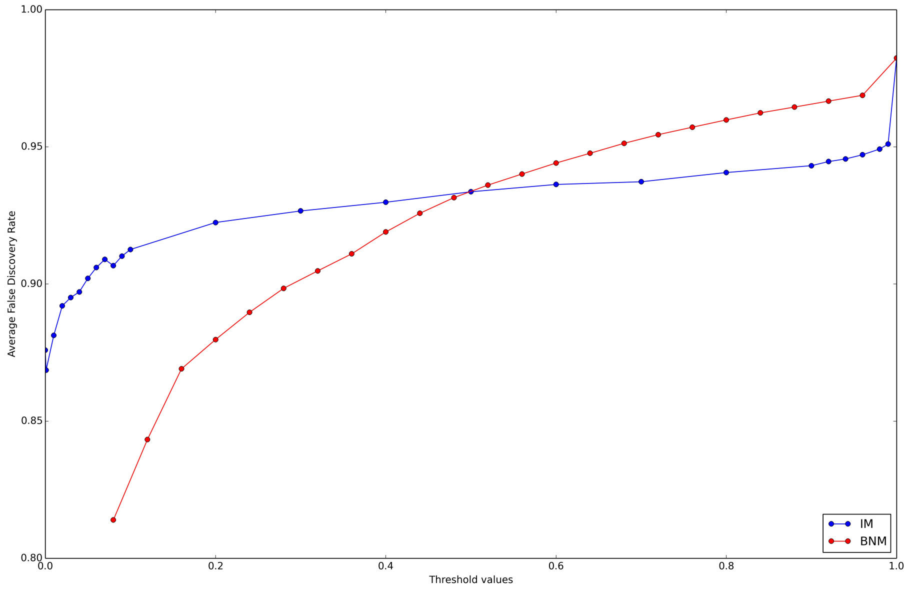

Figure S4: The average false discovery rate (y-axis) is the average over the FDRs within each of the 76 effect size groups in Fig. S3 at different P-value (for IM (blue)) or R-value (for BNM (red)) thresholds (x-axis).

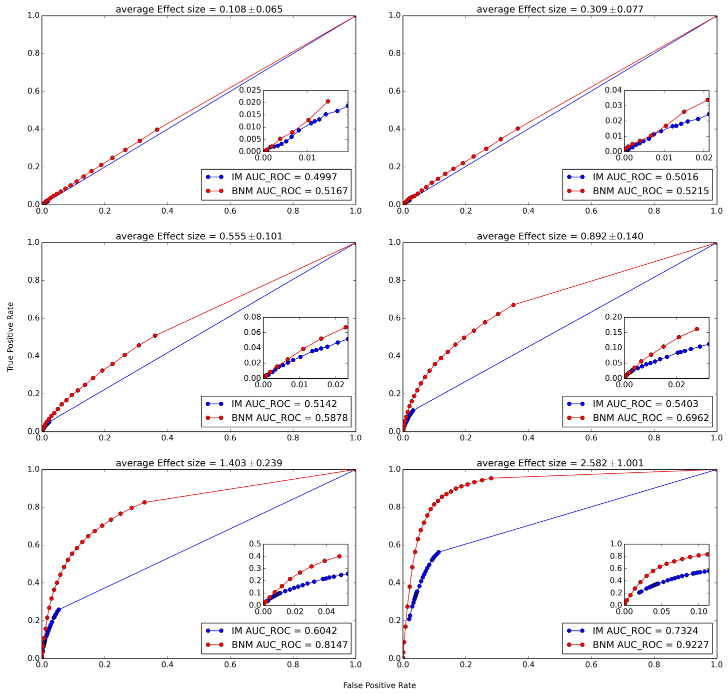

Figure S5: ROC curves for IM (blue) and BNM (red) within 6 groups of 3800 data points with average effect sizes 0.108 ± 0.065, 0.309 ± 0.077, 0.555 ± 0.101, 0.892 ± 0.140, 1.403 ± 0.239 and 2.582 ± 1.00, and with 2 Mb blocks.

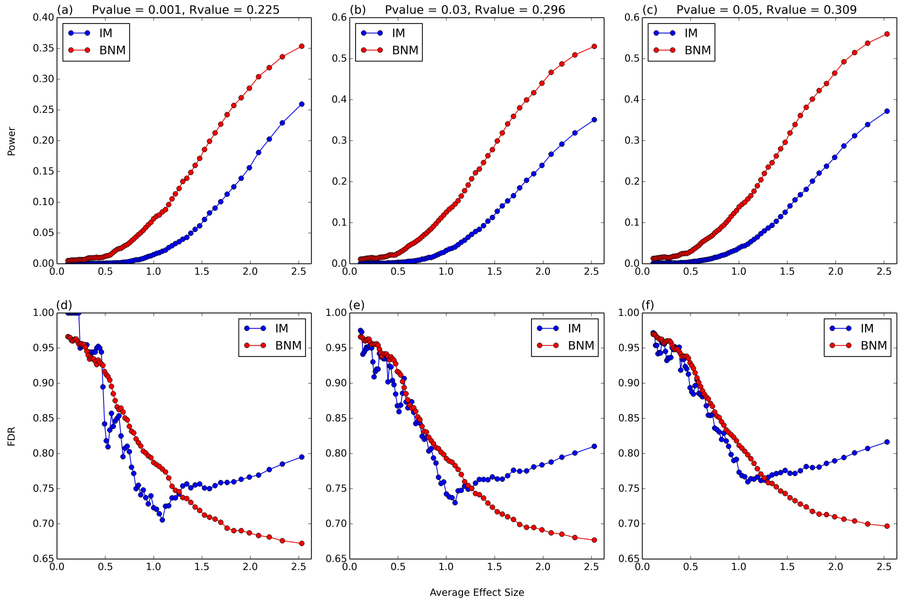

Figure S6: Power and FDR of the BNM algorithm (blue) and IM from the R/qtl package (red) with increasing effect sizes. Here we set 3 Mb blocks. Each point corresponds to the Power (a-b) or FDR (d-f) within a group of 4000 data points with an average effect size in the x-axis. We show the power and FDR at three P-value (for IM) and R-value (for BNM) thresholds: 0.001 and 0.225 (a, d), 0.03 and 0.296 (b, e), and 0.05 and 0.309 (c, f). These P-value, R-value pairs are matched so that they have the same FDR averaged over all points (see Fig. S7).

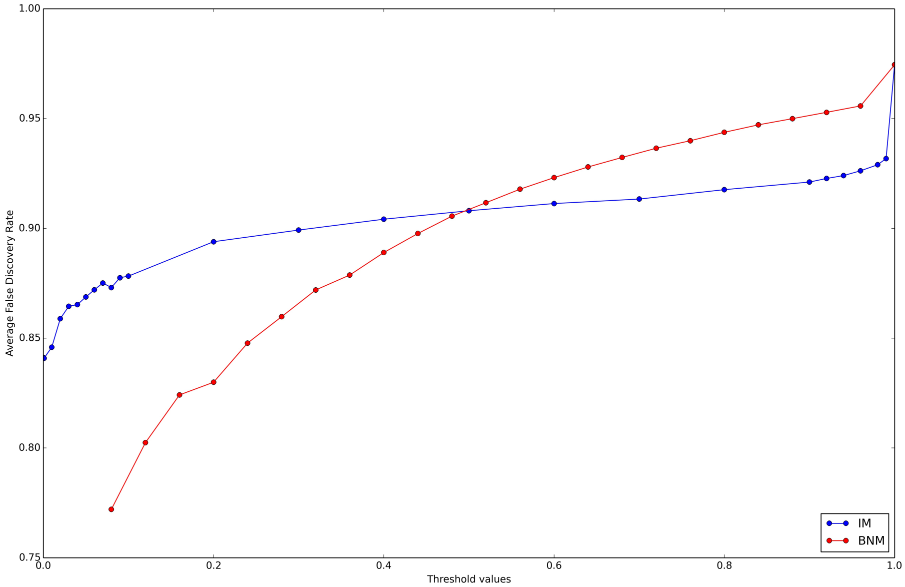

Figure S7: The average false discovery rate (y-axis) is the average over the FDRs within each of the 76 effect size groups in Fig. S6 at different P-value (for IM (blue)) or R-value (for BNM (red)) thresholds (x-axis).

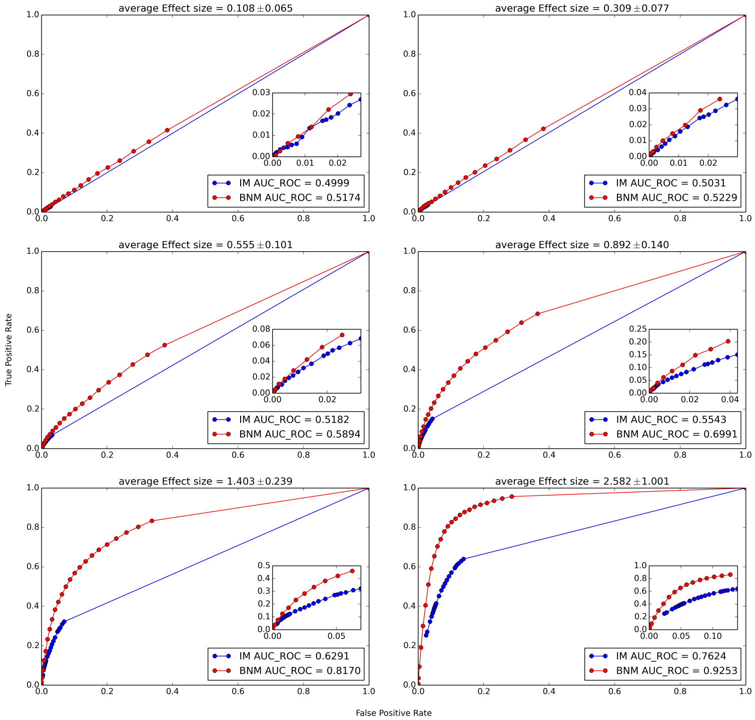

Figure S8: ROC curves for IM (blue) and BNM (red) within 6 groups of 3800 data points with average effect sizes 0.108 ± 0.065, 0.309 ± 0.077, 0.555 ± 0.101, 0.892 ± 0.140, 1.403 ± 0.239 and 2.582 ± 1.00, and with 3 Mb blocks.

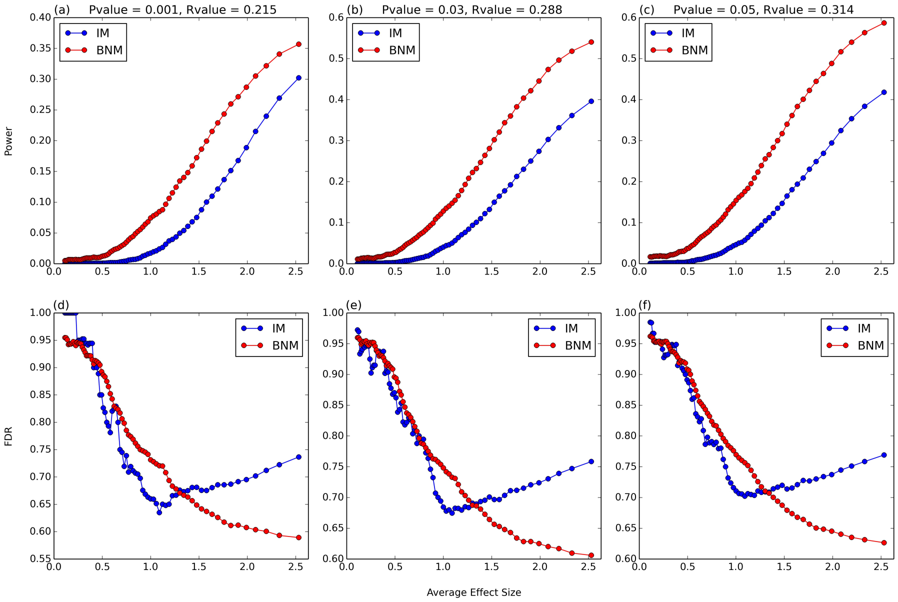

Figure S9: Power and FDR of the BNM algorithm (blue) and IM from the R/qtl package (red) with increasing effect sizes. Here we set 4 Mb blocks. Each point corresponds to the Power (a-b) or FDR (d-f) within a group of 4000 data points with an average effect size in the x-axis. We show the power and FDR at three P-value (for IM) and R-value (for BNM) thresholds: 0.001 and 0.215 (a, d), 0.03 and 0.288 (b, e), and 0.05 and 0.314 (c, f). These P-value, R-value pairs are matched so that they have the same FDR averaged over all points (see Fig. S10)

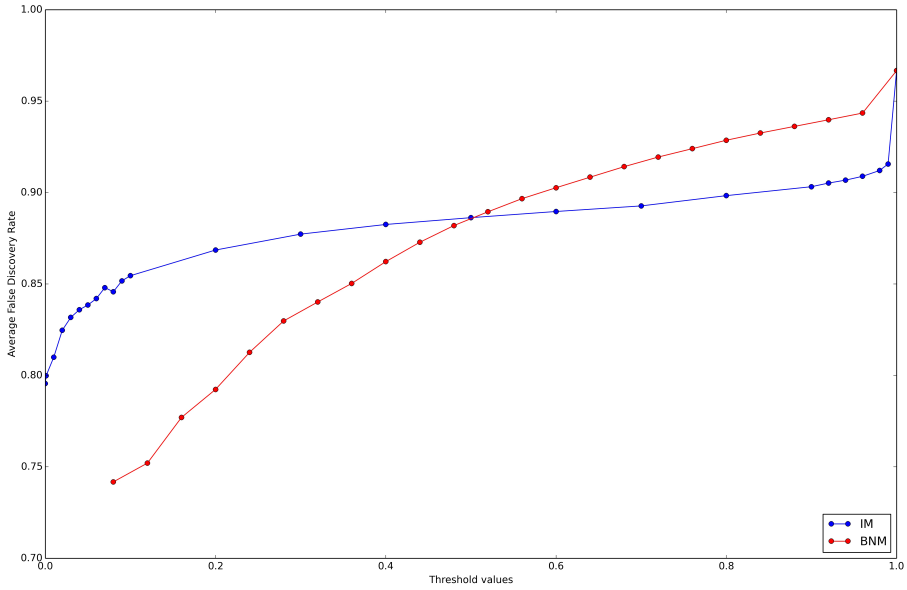

Figure S10: The average false discovery rate (y-axis) is the average over the FDRs within each of the 76 effect size groups in Fig. S9 at different P-value (for IM (blue)) or R-value (for BNM (red)) thresholds (x-axis).

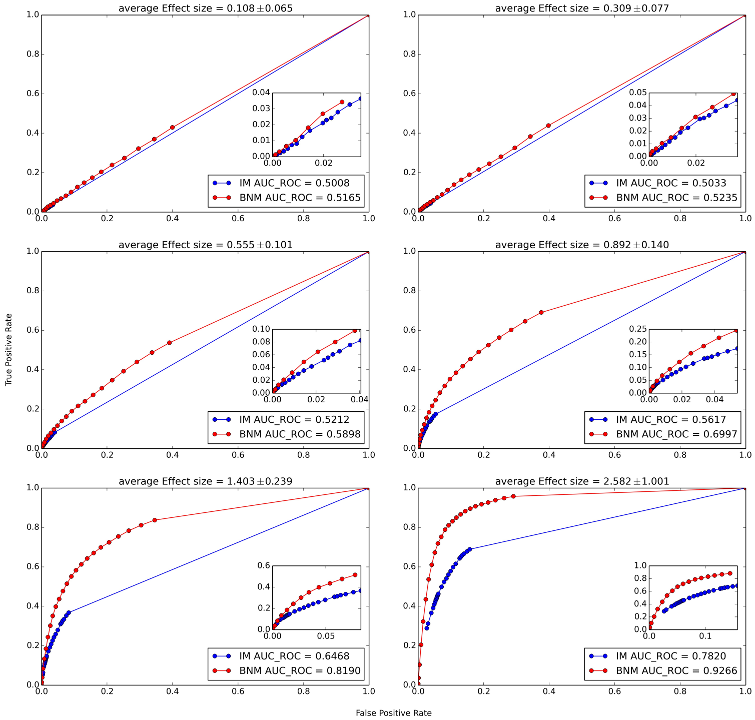

Figure S11: ROC curves for IM (blue) and BNM (red) within 6 groups of 3800 data points with average effect sizes 0.108 ± 0.065, 0.309 ± 0.077, 0.555 ± 0.101, 0.892 ± 0.140, 1.403 ± 0.239 and 2.582 ± 1.00, and with 4 Mb blocks.

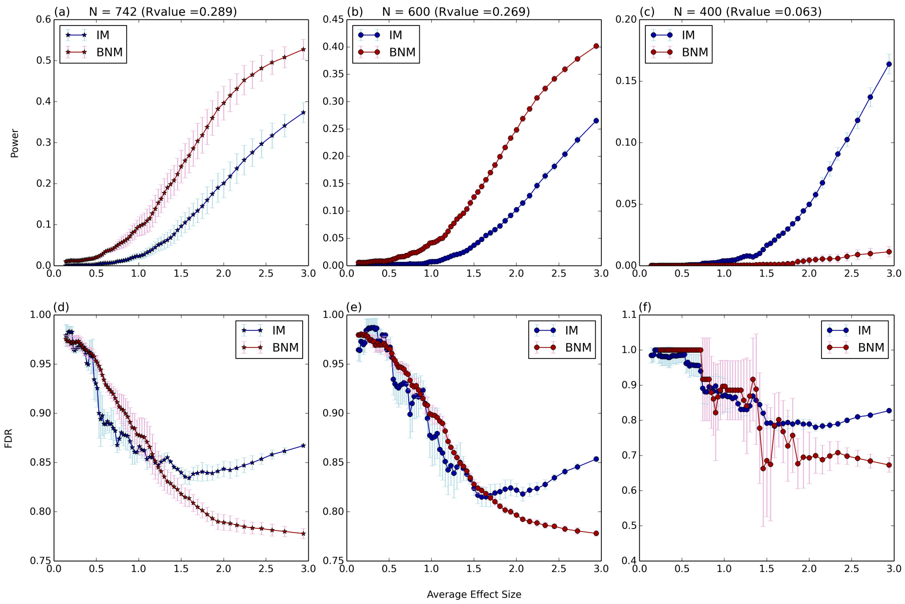

Figure S12: Power and FDR with 2 Mb blocks of the BNM algorithm (blue) and IM from the R/qtl package (red) with increasing effect sizes. Each point corresponds to the Power (a-c) or FDR (d-f) within a group of 2000 data points with an average effect size in the x-axis. We show the power and FDR at P-value = 0.05 (for IM) and the matching BNM R-value such that IM and BNM have the same FDR averaged over all points (see Fig. S13). In (a,d) we use all the mice (Nmice = 742) and three samples of 500 phenotypes from the 1000 simulated phenotypes; the FDR matching R-value = 0.362 (see Fig. S13a). In (b, e) we use three samples of randomly selected 600 mice out the 742 mice available; the FDR matching R-value = 0.492 (see Fig. S13b). In (c, f) we use three samples of randomly selected 400 mice out the 742 mice available; the FDR matching R-value = 0.281 (see Fig. S13c).The plots are the means over the three samples in each case, and the errorbars are the standard deviations from the mean in each case.

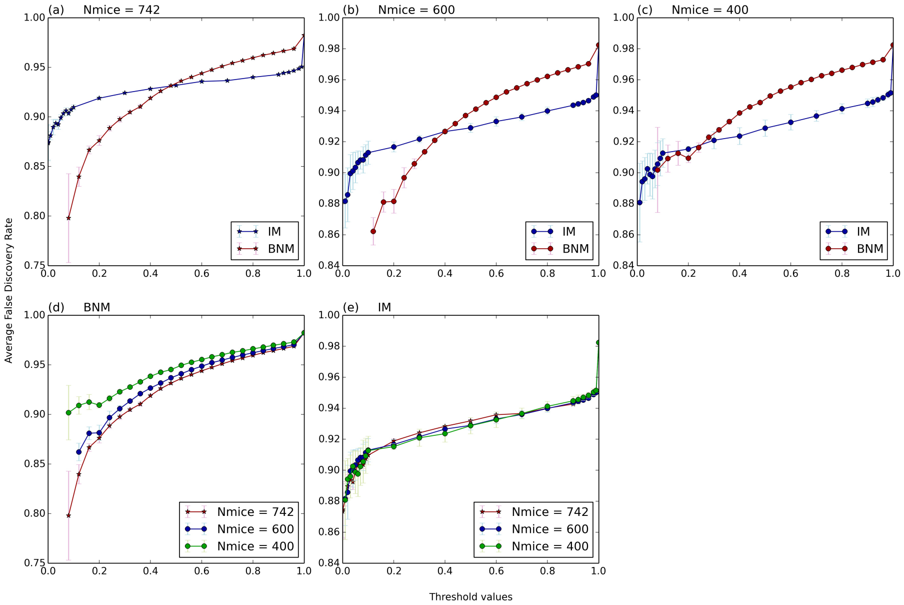

Figure S13: The average false discovery rates (y-axis) are the mean over the three samples of the average over the FDRs within each of the 76 effect size groups in Fig. S12 at different P-value (for IM (blue)) or R-value (for BNM (red)) thresholds (x-axis). In (a) we take the average FDR over the points in Fig. S12d for each of the three samples. The plot is the mean over the three samples and the errorbars are the standard deviation from the mean. Similarly we take the average over the points in Fig. S12e (b), and Fig. S12f (c). In (d) we replot all together the results for BNM (red plots in (a-c)). In (e) we replot all together the results for IM (blue plots in (a-c).

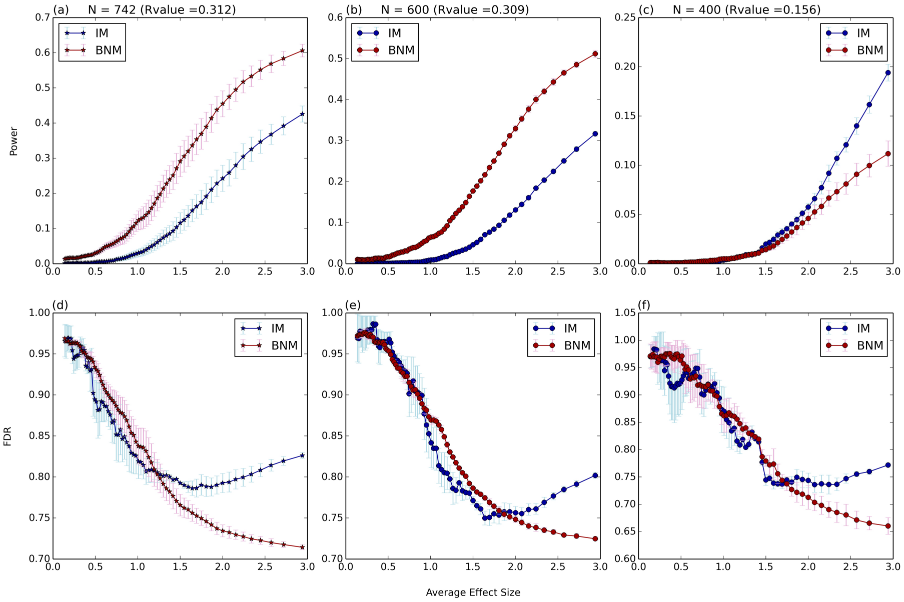

Figure S14: Power and FDR with 3 Mb blocks of the BNM algorithm (blue) and IM from the R/qtl package (red) with increasing effect sizes. Each point corresponds to the Power (a-c) or FDR (d-f) within a group of 2000 data points with an average effect size in the x-axis. We show the power and FDR at P-value = 0.05 (for IM) and the matching BNM R-value such that IM and BNM have the same FDR averaged over all points (see Fig. S15). In (a, d) we use all the mice (Nmice = 742) and three samples of 500 phenotypes from the 1000 simulated phenotypes; the FDR matching R-value = 0.362 (see Fig. S15a). In (b, e) we use three samples of randomly selected 600 mice out the 742 mice available; the FDR matching R-value = 0.492 (see Fig. S15b). In (c, f) we use three samples of randomly selected 400 mice out the 742 mice available; the FDR matching R-value = 0.281 (see Fig. S15c).The plots are the means over the three samples in each case, and the errorbars are the standard deviations from the mean in each case.

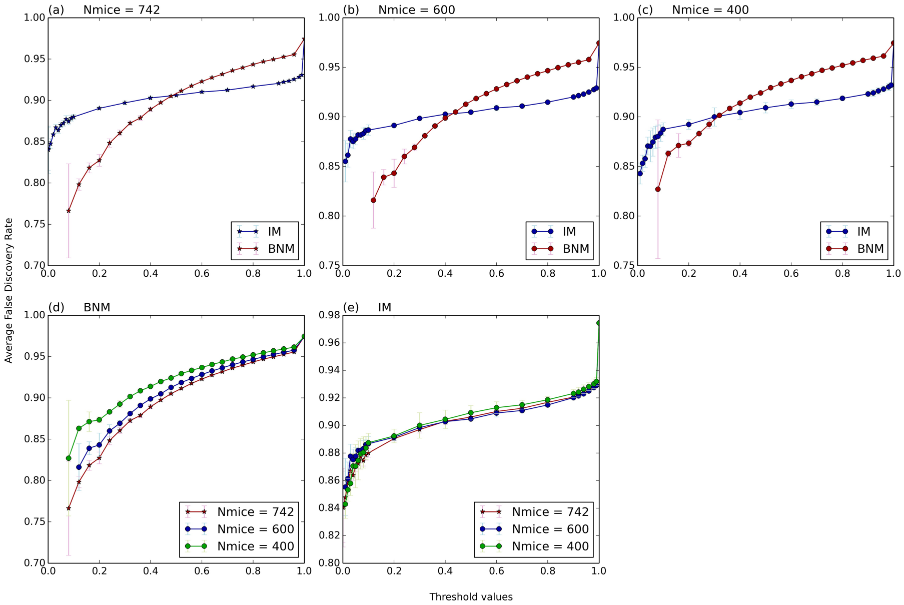

Figure S15: The average false discovery rates (y-axis) are the mean over the three samples of the average over the FDRs within each of the 76 effect size groups in Fig. S14 at different P-value (for IM (blue)) or R-value (for BNM (red)) thresholds (x-axis). In (a) we take the average FDR over the points in Fig. S14d for each of the three samples. The plot is the mean over the three samples and the errorbars are the standard deviation from the mean. Similarly we take the average over the points in Fig. S14e (b), and Fig. S14f (c). In (d) we replot all together the results for BNM (red plots in (a-c)). In (e) we replot all together the results for IM (blue plots in (a-c).

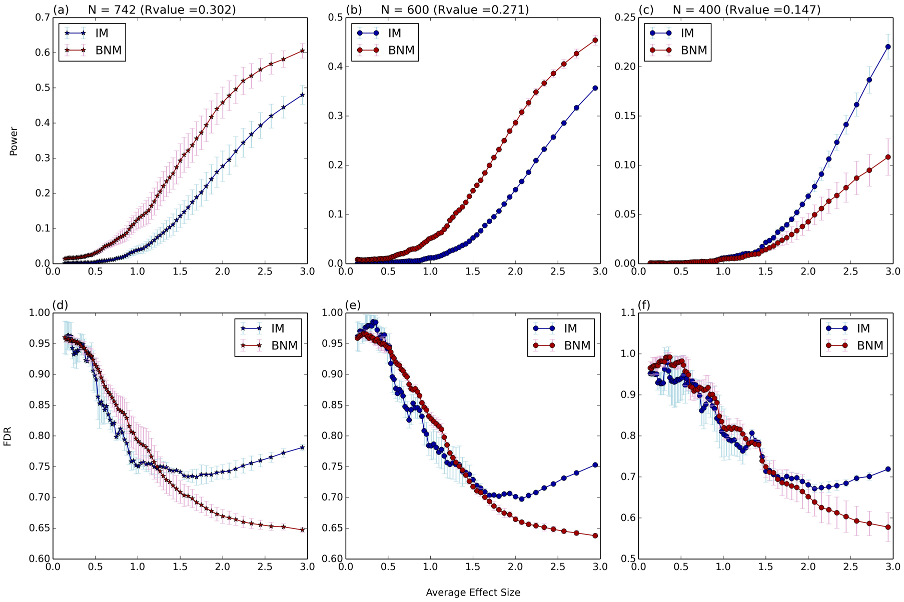

Figure S16: Power and FDR with 4 Mb blocks of the BNM algorithm (blue) and IM from the R/qtl package (red) with increasing effect sizes. Each point corresponds to the Power (a-c) or FDR (d-f) within a group of 2000 data points with an average effect size in the x-axis. We show the power and FDR at P-value = 0.05 (for IM) and the matching BNM R-value such that IM and BNM have the same FDR averaged over all points (see Fig. S17). In (a, d) we use all the mice (Nmice = 742) and three samples of 500 phenotypes from the 1000 simulated phenotypes; the FDR matching R-value = 0.362 (see Fig. S17a). In (b, e) we use three samples of randomly selected 600 mice out the 742 mice available; the FDR matching R-value = 0.492 (see Fig. S17b). In (c, f) we use three samples of randomly selected 400 mice out the 742 mice available; the FDR matching R-value = 0.281 (see Fig. S17c).The plots are the means over the three samples in each case, and the errorbars are the standard deviations from the mean in each case.

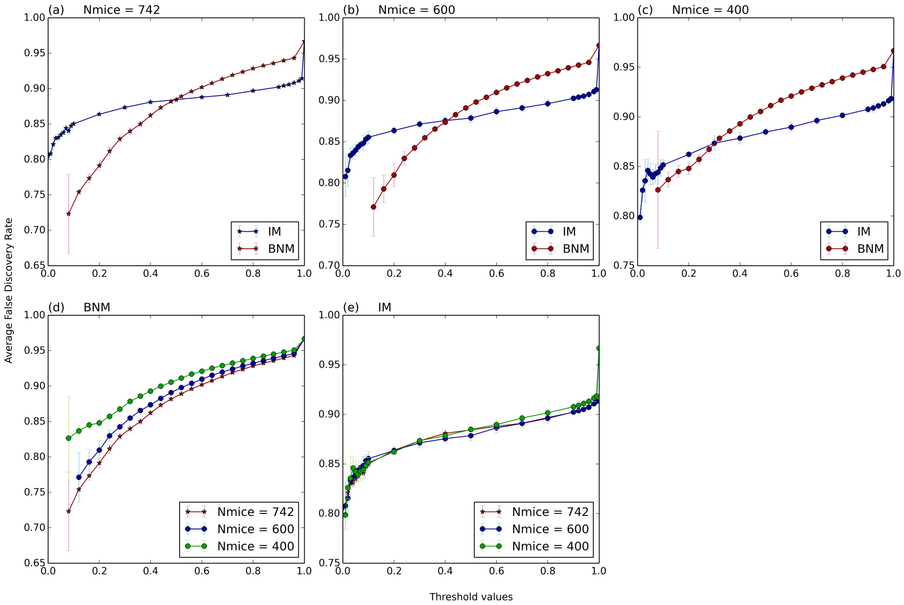

Figure S17: The average false discovery rates (y-axis) are the mean over the three samples of the average over the FDRs within each of the 76 effect size groups in Fig. S16 at different P-value (for IM (blue)) or R-value (for BNM (red)) thresholds (x-axis). In (a) we take the average FDR over the points in Fig. S16d for each of the three samples. The plot is the mean over the three samples and the errorbars are the standard deviation from the mean. Similarly we take the average over the points in Fig. S16e (b), and Fig. S16f (c). In (d) we replot all together the results for BNM (red plots in (a-c)). In (e) we replot all together the results for IM (blue plots in (a-c).

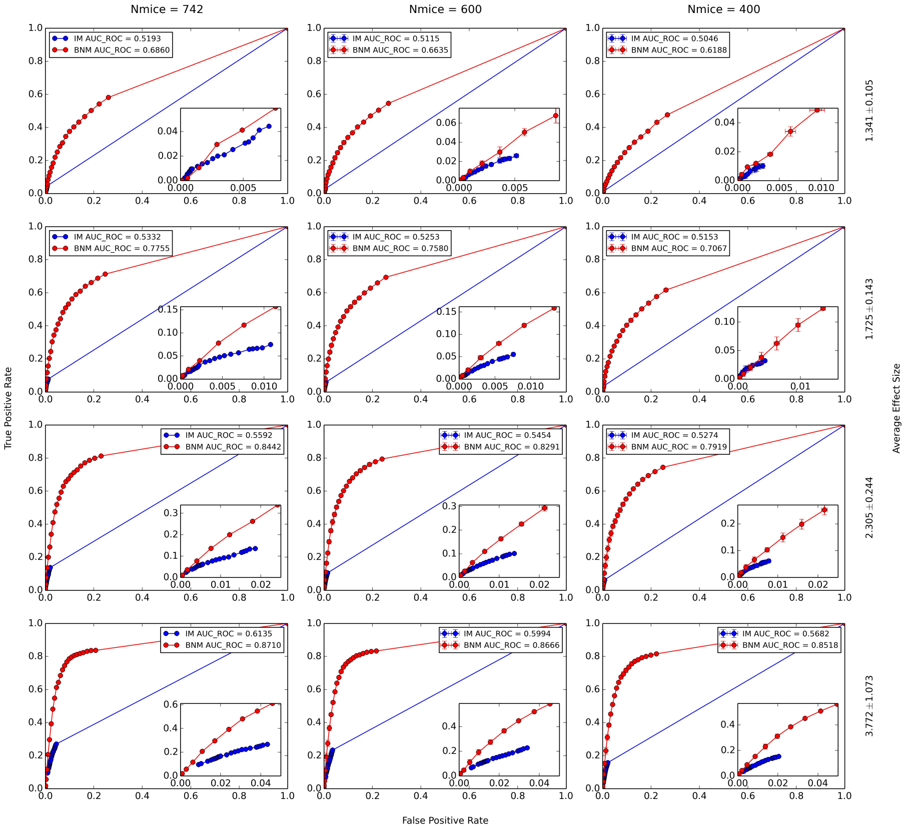

Figure S18: ROC curves for IM (blue) and BNM (red) within 4 groups of 950 data points with average effect sizes 1.314 ± 0.105, 1.725 ± 0.143, 2.305 ± 0.244 and 3.772 ± 1.073, for simulations with 500 phenotypes and 742 mice (left), 600 mice (center), and 400 mice (right). For the 600 and 400 mice the ROC curves are the averages over 3 samples of randomly selected mice and the error bars are standard deviations from the mean of the false discovery rate (horizontal) and true positive rate (vertical).

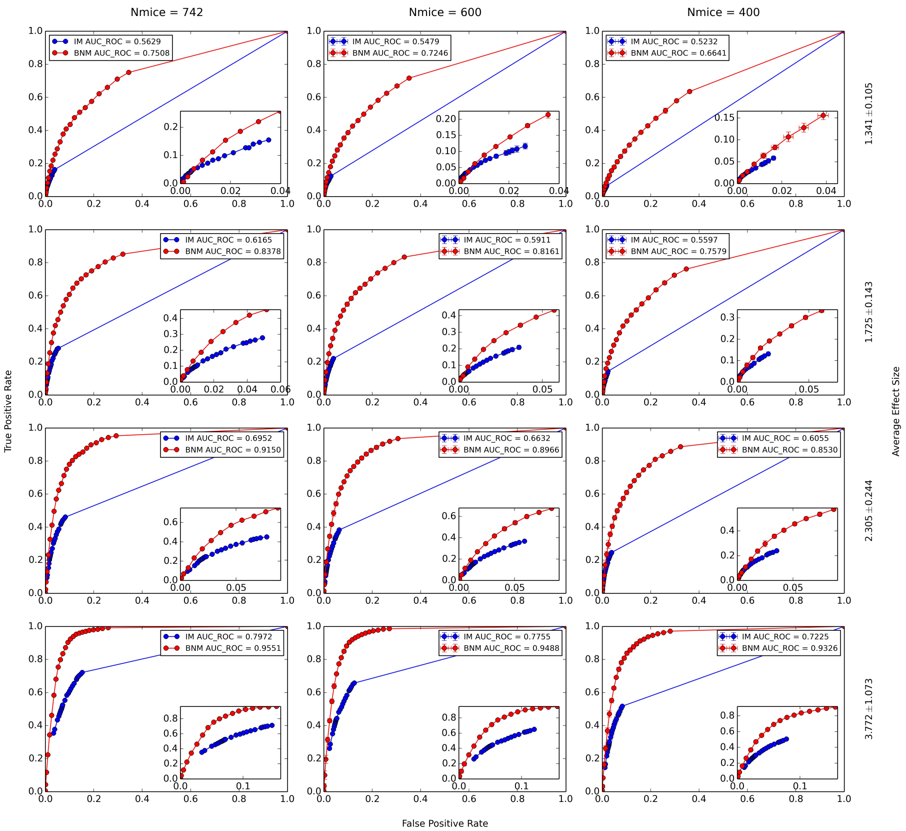

Figure S19: ROC curves for IM (blue) and BNM (red) within 4 groups of 950 data points with average effect sizes 1.314 ± 0.105, 1.725 ± 0.143, 2.305 ± 0.244 and 3.772 ± 1.073, and with 2 Mb blocks, for simulations with 500 phenotypes and 742 mice (left), 600 mice (center), and 400 mice (right). For the 600 and 400 mice the ROC curves are the averages over 3 samples of randomly selected mice and the error bars are standard deviations from the mean of the false discovery rate (horizontal) and true positive rate (vertical).

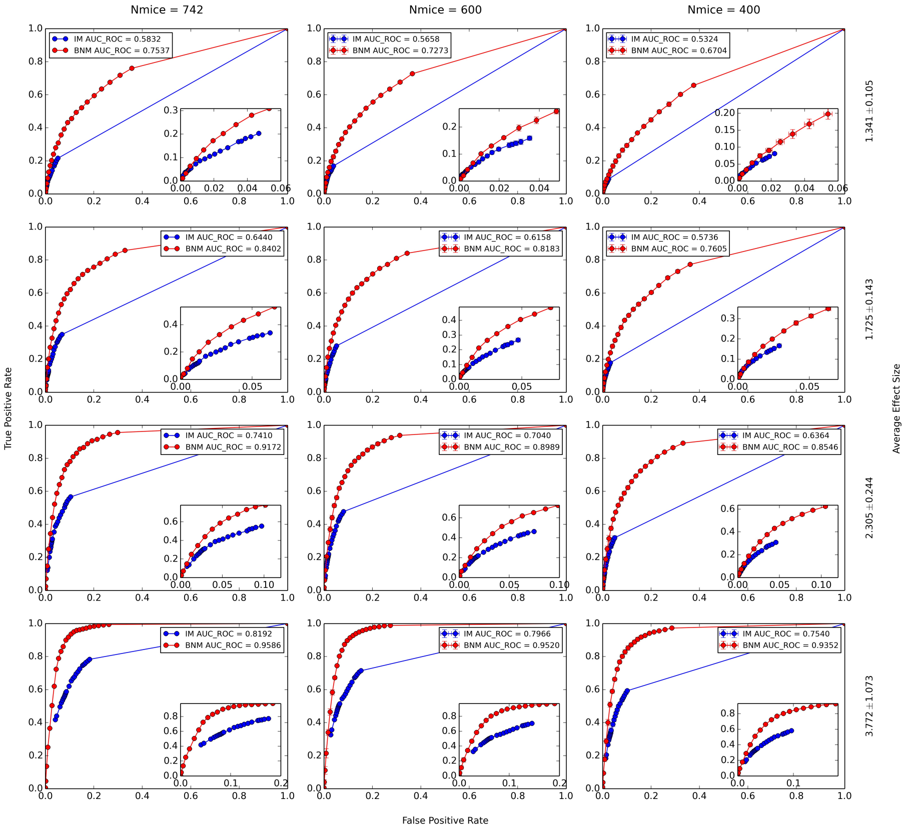

Figure S20: ROC curves for IM (blue) and BNM (red) within 4 groups of 950 data points with average effect sizes 1.314 ± 0.105, 1.725 ± 0.143, 2.305 ± 0.244 and 3.772 ± 1.073, and with 3 Mb blocks, for simulations with 500 phenotypes and 742 mice (left), 600 mice (center), and 400 mice (right). For the 600 and 400 mice the ROC curves are the averages over 3 samples of randomly selected mice and the error bars are standard deviations from the mean of the false discovery rate (horizontal) and true positive rate (vertical).

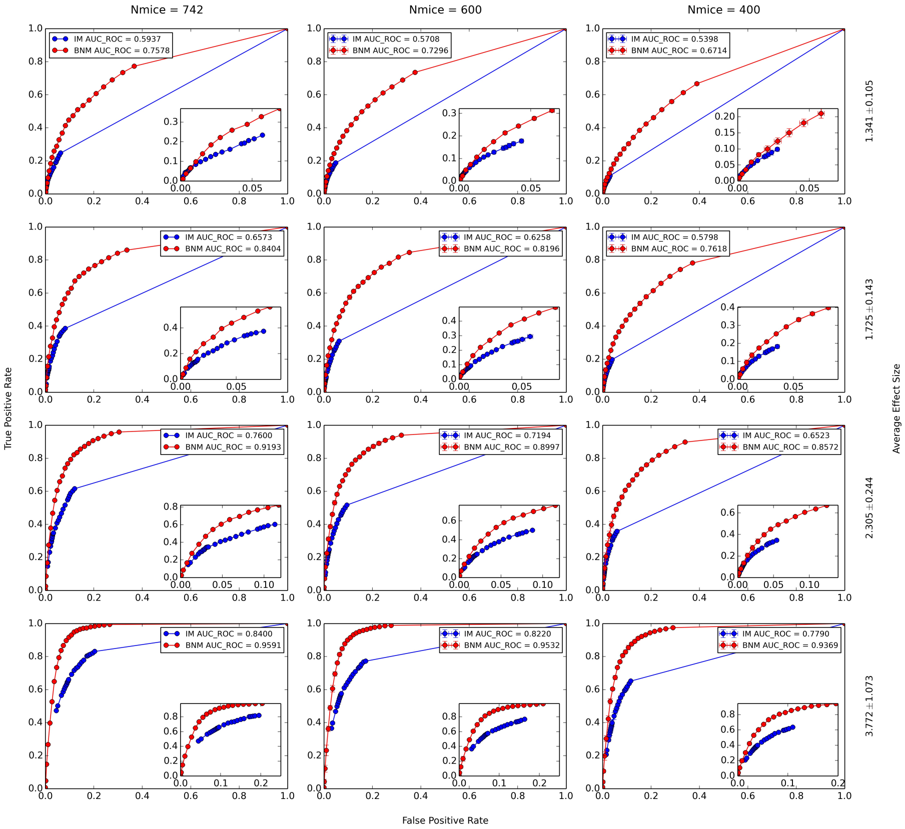

Figure S21: ROC curves for IM (blue) and BNM (red) within 4 groups of 950 data points with average effect sizes 1.314 ± 0.105, 1.725 ± 0.143, 2.305 ± 0.244 and 3.772 ± 1.073, and with 4 Mb blocks, for simulations with 500 phenotypes and 742 mice (left), 600 mice (center), and 400 mice (right). For the 600 and 400 mice the ROC curves are the averages over 3 samples of randomly selected mice and the error bars are standard deviations from the mean of the false discovery rate (horizontal) and true positive rate (vertical).
